# Robotic surgery versus conventional laparoscopy in colon cancer patients: a systematic review and meta-analysis

**DOI:** 10.1590/acb397224

**Published:** 2024-10-25

**Authors:** Giuliana Fulco Gonçalves, Pedro Vilar de Oliveira Villarim, Vitória Ribeiro Dantas Marinho, Clarissa Amaral Abreu, Luiz Henrique Moreira Pereira, Luiz Henrique Moreira Pereira, Sofia Emerenciano Gurgel, Amália Cínthia Meneses Rêgo, Kleyton Santos de Medeiros, Irami Araújo-Filho

**Affiliations:** 1 Instituto de Ensino, Pesquisa e Inovação Liga Contra o Câncer – Natal (RN) – Brazil.; 2 Universidade Potiguar – Department of Medicine – Natal (RN) – Brazil.; 3 Universidade Federal do Rio Grande do Norte – Department of Medicine – Natal (RN), Brazil.; 4 Universidade Federal do Rio Grande do Norte – Postgraduate Program in Health Sciences – Natal (RN), Brazil.; 5 Universidade Federal do Rio Grande do Norte – Department of Nursing – Natal (RN), Brazil.

**Keywords:** Colonic Neoplasms, Robotic Surgical Procedures, Colectomy

## Abstract

**Purpose:**

To compare robotic versus laparoscopic colectomies in colon cancer patients in general complications.

**Methods:**

Nine databases were searched for randomized controlled trials (RCT) investigating patients with colon cancer, submitted to robotic surgery (RS) compared to a laparoscopic (LC) approach. The risk of bias was assessed using RoB 2.0 tool, and certainty of the evidence was evaluated by Grading of Recommendations, Assessment, Development and Evaluation (GRADE). Data synthesis was performed using the software R. The meta-analysis of the included studies was carried out using the fixed-effects model (DerSimonian and Laird). Heterogeneity was measured using I2 analysis.

**Results:**

A total of four studies were used with 293 patients. Three studies were used in this comparative LC vs. RS when evaluating infection rates on surgical wound sites. The odds ratio (OR) appeared to be slightly favorable to LC (OR = 3.05; 95% confidence interval–95%CI 0.78–11.96). In the hospitalization rates analysis, two randomized controlled trials were used, and the mean differences slightly favored the RS (MD = -0.54; 95%CI -2.28–1.19). GRADE evaluation detected a serious risk of bias due to RCT format and RoB-2 concurred.

**Conclusion:**

Both types of procedures seem to have their own benefits, risks, and limitations. They seem close to equal in terms of postsurgical infection and hospitalization.

## Introduction

Colorectal cancer is the second most common type of neoplasm in the world and can be only cured through surgery, mainly colectomy^1^^–^ 3. Postoperative complications occur in more than 50% of the procedures, which negatively impact overall patient health and increase financial costs^4^. Therefore, a special focus in literature is needed to investigate the safest and most effective way to perform the procedure. Ongoing debates in the medical community propose changes in standardized procedures, comparing robotic and laparoscopic surgery^5^^–^ 7.

The evolution of robotic surgery is considered a major innovation in modern medicine, as it provides alternative surgeries for multiple scenarios^8^. Although some studies select it as more time-consuming and expensive than laparoscopy, it could provide faster recovery of bowel function, shorter hospital length of stay, improvement in blood loss, rates of complications and wound site infection, when compared to the common laparotomy^9^^–^ 11. However, other technical disadvantages exist, such as limited range of motion of instruments and related loss of dexterity, fixed instrument tips, and an inadequate visual field associated with an unstable view of the camera and traction of the assistant^12^^–^ 14.

Currently, the laparoscopic approach is the gold-standard treatment for colon cancer^15^. Nevertheless, considering the new evidence on robotic surgery, it should be ensured that it remains the strategy that enables safer, more favorable, cost-effective outcomes for patients^16^^–^ 18. Colectomy is a medium to large operation, depending on the tumor staging^19^. Thus, some of the postoperative complications, such as wound infection, sepsis, and malnutrition, increase the length of hospital stay and the morbidity and mortality of operated patients^20^^–^ 22. However, two of the most worrying and difficult-to-manage complications are infection and digestive fistulas^23^.

Infections correspond to the leading cause of hospitalization and post-surgical complication, especially when sepsis is accounted for. Due to the interference in homeostasis, this condition and its treatment represent a challenge^24^. For most elective procedures, mortality rates are low, below 2%. However, in patients that develop digestive fistulae, this value increases from anywhere to 6 up to 48%, even with advanced treatment, especially due to conversion to sepsis and intensive care unit. Cancer patients are even more vulnerable to all these complications^25^^–^ 27.

A lot of data regarding postoperative complications of robotic surgery is unknown^16^^,^ 17. Regarding that, a detailed understanding of the prevalence of its implications among cancer patients is necessary, as they represent a vulnerable group subject^10^.

Therefore, this study aimed to compare outcomes in laparoscopic colectomies with colectomies performed by robotic surgery in patients with colon cancer undergoing curative oncologic surgery.

## Methods

This review was conducted in accordance with the Preferred Reporting Items for systematic reviews and meta-analysis (PRISMA), and the Cochrane Collaboration reporting items for systematic reviews and meta-analysis, with a review protocol developed and registered on Prospective Register of Systematic Reviews (PROSPERO reference CRD42021295313), and was previously published in a scientific journal.

### Review question

- Will robotic surgery improve infection outcomes in these patients?
- What are other complications associated with both types of colectomies?

The questions were formulated based on the PICOS framework, and the elements were as follow:

- Population: patients with colon cancer.
- Intervention: robotic surgery.
- Comparison: conventional laparoscopic colectomy.
- Outcome: surgical infection rates, sepsis, length of hospital stays, mortality.
- Study design: randomized clinical trials.

### Eligibility criteria

The systematic review included randomized clinical trials that compared outcomes regarding laparoscopic and robotic colectomy in colon cancer patients. Studies should have adult patients (age > 18 years old) with colon cancer. No language or time restrictions were applied. Case series and reports, cohorts and pre-clinical trials were excluded from this review, as well as studies that did not meet the inclusion criteria.

### Outcome measures

This review evaluated many factors, such as:

- Postoperative infection rates: various studies in the systematic review assessed postoperative infection rates, meaning patients that contracted any sort of wound infection pertaining to their operation or postoperative period;
- Sepsis: the studies evaluated if the infections were systemic and life threatening;
- Morbimortality: the studies appraised other risks the patients were subjected during or after the procedure;
- Length of hospital stay: the randomized controlled trials (RCT) rated the duration of hospitalization of each procedure.

### Data sources and searches

A data search was conducted on multiple databases using Medical Subject Heading (MeSH) search terms, text words, and keywords based on each database characteristic focusing on synonyms for colonic fistulas and robotic surgery for colectomy in oncologic patients. Databases were PubMed, Embase, Scopus, Web of Science, Science Direct, Latin American and Caribbean Health Sciences Literature (LILACS), Cochrane Central Register of Clinical Trials, CINAHL, and clinical trial databases (www.trialscenter.org, www.controlled-trials.com, www.clinicaltrials.gov).

The MeSH search was: ((Colonic Neoplasm OR Colonic Cancer OR Cancer of Colon) AND (Laparoscopic Colectomy OR Right Colectomy OR Sigmoidectomy OR Hemicolectomy) AND (Robotic Surgical Procedures OR Robot OR Robot-assisted) AND (Digestive System Fistula OR Intestinal Fistula OR Infection OR Sepsis OR Mortality OR Length of Hospitalization OR Malnutrition)). The searches were synthesized through systematic review search tool Rayyan^26^^,^ 27.

### Data extraction

For each eligible study, data extraction was conducted independently by two reviewers, using a standardized data extraction form. Any discrepancies between the two reviewers were resolved through discussion and consensus with a third reviewer. The following information was extracted from each included study: author(s), publication year, study design, sample size, participant characteristics (sex, age), intervention details (resection site, type of intervention), control group (details such as ASA score, body mass index, infection rates, sepsis) outcome measures, and relevant results. The primary outcome was surgical wound infection rate, and the secondary outcome was length of hospital stay.

### Risk of bias assessment

Two reviewers independently assessed the quality of the studies using the Cochrane Collaboration’s Risk of Bias Tool for RCTs^27^. Each study was evaluated for potential bias in key domains such as random sequence generation, allocation concealment, blinding of participants and personnel, blinding of outcome assessment, incomplete outcome data, and selective reporting. Any disagreements in the assessment were resolved through discussion with a third reviewer.

### GRADE certainty of evidence

Quality of evidence was assessed using the Grading of Recommendations, Assessment, Development and Evaluation (GRADE) approach for primary outcomes and serious adverse events^28^^–^ 30.

### Data synthesis

A meta-analysis was performed using the R Project for Statistical Computing software (version R-4.3.1)^24^. For each included randomized clinical trial, continuous outcomes were presented as mean ± standard deviation, mean differences (MD), standardized mean differences (SMD), or hazard ratios (HR), with inverse-variance fixed-effect and dichotomous outcomes as odds ratios (ORs) with Mantel-Haenszel random-effects analysis and 95% confidence intervals (95%CI) for all outcome measurements. Cochrane’s Q test was applied to quantify heterogeneity among studies, and with the I2 test assessed for inconsistency^28^. With I2 intervals between 0–50%, acceptable heterogeneity was present.

## Results

**Figure 1:**
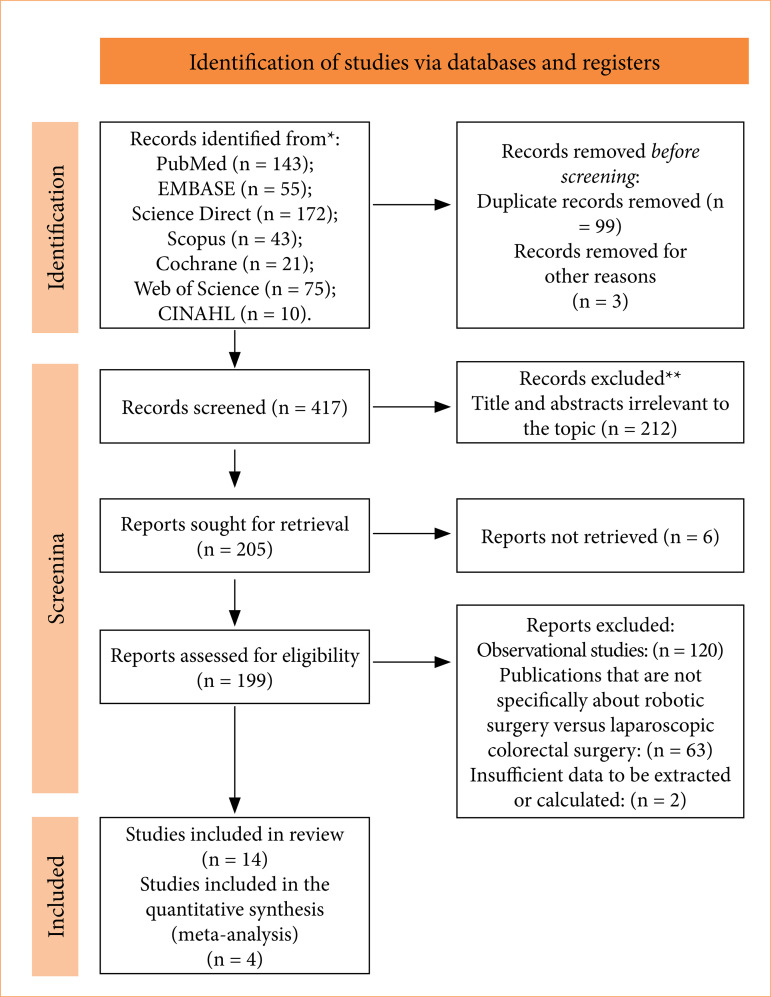
Flowchart for study selection. PRISMA flow diagram for systematic review and meta-analysis.

Source: Elaborated by the authors.

The electronic search identified a total of 498 studies before screening. After running duplicate removal, 99 records were removed, with other three being excluded in this process. Out of the 396 articles left, 212 titles and/or abstracts were proven irrelevant to the study. Then, 205 reports were sought for retrieval, with six not being retrieved. Then, 120 were excluded for being observational studies, 63 for not having laparoscopic surgery and robotic surgery in colorectal surgery as their main topic, and two were excluded for their data being insufficient to be extracted or calculated. After reviewing titles, abstracts, and full texts, 14 studies met the inclusion criteria and were selected to compose the review, and four were used to generate two meta-analyses^20^^–^ 24 (Fig. 1).

### Study characteristics

The studies included in the meta-analysis were randomized controlled trials published between 2012 and 2021, encompassing colon cancer populations subjected to the comparison of laparoscopic colectomy (LC) with robotic colectomy (RC) (Table 1). The sample sizes of the individual studies ranged from 70 to 97 participants, with a total of 293 participants across all studies. The mean of ages ranged from 52 to 78.

**Table 1:** Characteristics of the studies included in the systematic review.

Authors	Year	Country	Design	N Patients	Mean age
Park et al.^20^	2018	South K	ECR	RC 35/ LC 35	RC 62.8 / LC 66.5
Park et al.^21^	2012	South K	ECR	RC 35 / LC 35	RC 62.8 / LC 66.5
Yozgatli et al.^22^	2019	Turkey	ECR	RC 35 / LC 61	RC 65 ± 13 / LC 65 ± 13
Rattenborg et al.^23^	2021	DEN	ECR	RC 57 / LC 40	NA
Bednarski et al.^24^	2019	US	ECR	RC 21 / LC 9	NA

Source: Elaborated by the authors.

### Qualitative analysis

Among the selected RCTs, only four studies provided specific data on resection site, three clinical trials specified the ASA score of the patients, three on body mass index (BMI), three on infection, two on sepsis, three on mortality, and only two evaluated the mean hospitalization length of patients.

Most of the resections with both types of procedures seemed to include either the transverse or ascendent colon. No studies evaluated fistula outcomes, which was originally the main purpose of the review protocol. The trials that specified patient sex had more female than male participants. All patients were older than 60 years old. A tendency for patients with a higher ASA (II-III) to be placed under the robotic procedure was observed. Mean BMI was from 23.8 to 29. Only one patient presented sepsis. No deaths occurred during the trials or their follow-ups. Malnutrition indexes were not evaluated on any studies.

### Infection rates in robotic surgery vs. laparoscopic

**Figure 2:**
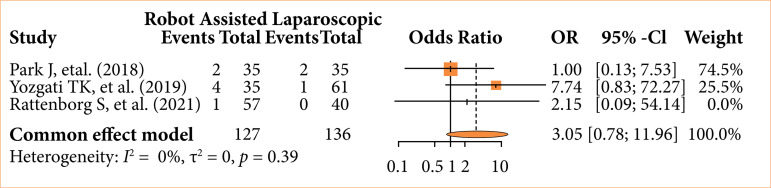
Forest plot for surgical infection rates.

Source: Elaborated by the authors.

Meta-analysis was used to analyze the combination of different studies. Each outcome was analyzed independently, with a total of three studies used in this comparative of LC vs. RC when evaluating infection rates on surgical wound sites. The OR appeared to be favorable to laparoscopic surgery (OR = 3.05; 95%CI 0.78–11.96), but it did not reach statistical significance. For this outcome, a fixed effect model was considered. A total of seven patients out of 127 had postsurgical wound infection after the robotic procedure versus three out of 136 after the laparoscopic procedure. After sensitivity analysis, no significant statistical difference was found between the studies, as shown in the forest plot (Fig. 2).

### Hospitalization rates in robotic surgery versus laparoscopic

**Figure 3:**
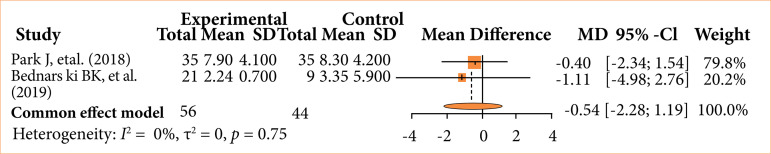
Forest plot for mean length of hospital stay.

Source: Elaborated by the authors.

In the hospitalization rates meta-analysis, two randomized controlled trials were used. The MD slightly favored the robotic approach (MD = -0.54; 95%CI -2.28–1.19). However, no statistically significant difference was found in this outcome (Fig. 3).

### Risk of bias

The risk of bias in each trial is shown in Table 2. In general, all the studies had a degree of risk of reporting bias. This is because, concerning the nature of the procedures, allocation concealment and blinding of all participants and personnel are not a possibility.

**Table 2:** Risk of bias.

Study/Year Reference	Random sequence generation	Allocation concealment	Blinding of participants and personnel	Blinding of outcome assessment	Incomplete outcome data assessed	Free of selective reporting	Free of other bias
Park et al.^20^/2018	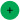	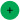	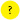	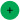	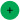	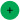	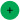
Park et al.^21^/2019	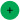	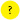	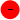	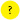	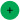	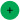	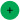
Yozgatli et al.^22^/2021	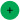	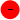	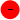	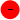	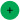	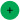	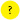
Rattenborg et al.^23^/2019	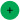	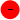	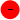	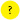	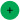	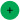	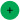

Source: Elaborated by the authors.

### Certainty of evidence

Additional digital content (Table 3) presents the quality and certainty of evidence for each outcome assessed in the clinical trials. The primary outcome, postoperative infection, and the other meta-analysis outcome were classified as having a low certainty of evidence, according to the GRADE guidelines.

**Table 3:** Grading of Recommendations, Assessment, Development and Evaluation (GRADE) quality of evidence.

Certainty assessment								N of patients			Effect		Certainty	Importance
N of studies	Study design	Risk of bias	Inconsistency	Indirectness	Imprecision	Other considerations		Robotic surgery	Laparoscopic surgery		Relative (95%CI)	Absolute (95% I)		
**Postsurgical infection**														
3	randomised trials	serious^a^	not serious	not serious	not serious	none		7/127 (5.5%)	3/136 (2.2%)		**OR 3.05** (0.78–11.96)	42 more per 1,000 (from 5 fewer to 190 more)	⨁⨁⨁◯ Moderate	IMPOR-TANT
**Length of hospital stay**														
2	randomised trials	serious^a^	not serious	not serious	not serious	none		56	44		-	**MD 0.54 SD lower** (2.28 lower to 1.19 higher)	⨁⨁⨁◯ Moderate	IMPOR-TANT

95%CI: 95% confidence interval; MD: mean difference; SD: standard deviation; OR: odds ratio. Source: elaborated by the authors.

## Discussion

Despite all the advances towards developing surgical techniques, the potential of the robotic surgery field has not been fully reached. A limited number of clinical trials evaluated the comparison between LC and RC in colon cancer patients^20^^–^ 24.

The studies did not yield their full capability pertaining to outcome measures that could be explored. Malnourishment, fistula, surgical dehiscence, and intraoperative occurrences were all factors in which the data was not retrieved from any clinical trial and could help indicate which procedure is more adequate, particularly settings, for these groups of colon cancer patients^14^^,^ 25.

Originally, this review’s protocol aimed to include fistulization as a main outcome, however, during study selection, it was noticed no randomized clinical trial had it documented as an outcome.

Only one of the trials included a long-term follow-up^21^^,^ 29, possibly because of how recent most of the trials are. It was possible to retrieve information for the infection rates and hospital length of stay. Thus, these becoming the main outcomes of the meta-analysis. Other outcomes were analyzed^30^. However, their results were incompatible with meta-analysis statistics, and therefore could only be presented as qualitative results.

With the infection rates outcome, no statistically significant difference was found. It was found that more surgical wound infection could be linked to the robotic approach. However, more studies, with a larger number of patients, would be necessary in order to determine that.

The hospitalization length was greater on the laparoscopic approach, meaning patients could remain in the hospital for a longer period if they were subjected to it. No statistical significance was found on this meta-analysis result^26^.

This review could not determine which strategy was more cost-effective. Most patients scored ASA I, but a larger percentage of patients with higher ASA scores were subjected to RC rather than LC, which could indicate a reason why the infection rates worsened^27^^,^ 28.

Only one case of sepsis was reported in all the procedures. Because of that, this result could not be included in the meta-analysis, even though the data was retrieved by the authors. The same happened to mortality rates. No patients died during or in the immediate follow-up of any procedure. Therefore, this result could not be analyzed by the meta-analysis.

Limitations

The present review and meta-analysis have some limitations. Some trials only featured a limited number of patients. Additionally, the nature of surgical procedures prevented the research from being completely blinded because, despite randomization, the surgeon would be aware of the surgery being performed and on which patient. Additionally, it makes sense that the given results are restricted to a brief follow-up time. High heterogeneity in the studies included in the evaluation as a result of the absence of standardization for evaluating the outcomes was another factor that affected the projected outcome.

It was also noticed that the absence of the evaluation of certain outcomes that were at aim in the review protocol could present a potential limitation. To reduce these limitations, several strategies were adopted. To minimize the risk of bias, the meta‐analysis was restricted to randomized clinical trials, and the quality of the included studies was reported using a validated scale. The authors were contacted, and reports not published in PubMed were obtained to minimize the risk of missing relevant studies. To ensure better certainty of the outcomes, a large sample size was used in the present systematic review.

## Conclusion

Both types of procedures seem to have their own benefits, risks, and limitations. From these RCTs alone, they are close to equal in terms of postsurgical infection and hospitalization. More studies, with larger patient samples, are necessary to determine if a statistical difference could be found between them. Moreover, considering the natural risk of bias these types of trial determine, larger samples and more information should be collected, and other outcomes should be accounted for.

## Data Availability

All data sets were generated or analyzed in the current study.
